# Impact of induction chemotherapy with intermediate-dosed cytarabine and subsequent allogeneic stem cell transplantation on the outcome of high-risk acute myeloid leukemia

**DOI:** 10.1007/s00432-021-03733-0

**Published:** 2021-07-23

**Authors:** Maximilian Fleischmann, Ulf Schnetzke, Jochen J. Frietsch, Herbert G. Sayer, Karin Schrenk, Jakob Hammersen, Anita Glaser, Inken Hilgendorf, Andreas Hochhaus, Sebastian Scholl

**Affiliations:** 1 Klinik Für Innere Medizin II, Abteilung Hämatologie Und Internistische Onkologie, Universitätsklinikum Jena, Am Klinikum 1, 07747 Jena, Germany; 2 4. Medizinische Klinik, HELIOS Klinikum Erfurt, Nordhäuser Straße 74, 99089 Erfurt, Germany; 3 Institut Für Humangenetik, Universitätsklinikum Jena, Am Klinikum 1, 07747 Jena, Germany

**Keywords:** High-risk AML, Induction chemotherapy, Allo-HSCT, RIC, CPX-351, MDS

## Abstract

**Background:**

Acute myeloid leukemia (AML) with antecedent hematological disease (s-AML) and treatment-related AML (t-AML) predicts poor prognosis. Intensive treatment protocols of those high-risk patients should consider allogeneic stem cell transplantation (allo-HSCT) in first complete remission (CR). Despite allo-HSCT, relapse rate remains high. Induction chemotherapy with liposomal cytarabine and daunorubicin (CPX-351) has been approved for patients with AML with myeloid-related changes (AML-MRC) or t-AML based on improved survival and remission rates compared to standard 7 + 3 induction.

**Patients and methods:**

110 patients with newly diagnosed s-AML or t-AML at a university hospital were analyzed retrospectively. Median age was 62 years (24–77 years). A total of 65 patients with s-AML after MDS (59%) and 23 patients (20.9%) with t-AML were included. Induction chemotherapy consisted of intermediate-dosed cytarabine (ID-AraC) in combination with idarubicin (patients up to 60 years) or mitoxantrone (patients over 60 years). In patients subsequently undergoing allo-HSCT, reduced conditioning regimens (RIC) were applied prior to transplantation in 47 of 62 patients (76%).

**Results:**

Induction chemotherapy with ID-AraC resulted in an overall response rate of 83% including complete remission (CR/CRi) in 69 patients (63%) with a low rate of early death (2.7%). Most relevant non-hematologic toxicity consisted of infectious complications including sepsis with need of intensive care treatment in five patients (4.5%) and proven or probable invasive fungal disease in eight patients (7.2%). Relapse-free survival (RFS), event-free survival (EFS) and overall survival (OS) of the whole cohort were 19 months (0–167), 10 months (0–234) and 15 months (0–234), respectively (*p* < 0.0001). A significant improvement of OS was observed in patients who underwent allo-HSCT compared to those without subsequent allo-HSCT: 9 vs. 46 months, *p* < 0.0001. Rate of transplantation-related mortality (TRM) in the early phase post allo-HSCT was low (0.9% at day 30 and 1.8% at day 90, respectively). RIC conditioning results in OS rate of 60% after 60 months post allo-HSCT (median OS not reached).

**Conclusion:**

S-AML and t-AML patients receiving induction chemotherapy with intermediate-dosed cytarabine showed satisfactory response rate and consolidation therapy with allo-HSCT after full or reduced-intensity conditioning further improved survival in these patients with similar outcome as reported for CPX-351.

**Supplementary Information:**

The online version contains supplementary material available at 10.1007/s00432-021-03733-0.

## Introduction

Incidence of secondary AML (s-AML) following myelodysplastic syndrome (MDS) is increasing with age and risk of transformation depends on stage of MDS as defined by percentage of blasts and genetic aberrations. Furthermore, AML with myeloid-related changes (AML-MRC) can be diagnosed in the absence of MDS history according to the WHO 2016 classification (Arber et al. 2016).

AML with an antecedent hematological disease (s-AML) is associated with unfavorable cytogenetic aberrations (e.g., complex karyotype) (Ostgård et al. 2010). A monosomal karyotype has a negative prognostic impact not only in patients with complex cytogenetic aberrations but also in AML with MDS-related karyotype anomalies (Kayser et al. 2012). Large population-based AML registries confirm a lower rate of complete remission following induction chemotherapy and worse outcome in patients with s-AML (Hulegårdh et al. 2015). During the transformation of MDS to s-AML the majority of patients gain additional cytogenetic or molecular genetic aberrations (e.g., FLT3 mutations) and distinct mutation patterns can be detected during progression of MDS to s-AML (Flach et al. 2011; Fernandez-Mercado et al. 2012).

The presence of TP53 mutations in AML patients with complex cytogenetic aberrations can regularly be observed in combination with a monosomal karyotype and is associated with age, a reduced response rate and worse outcome (Rücker et al. 2012). In patients with complex karyotype and either TP53 or RAS mutations even allogeneic stem cell transplantation (allo-HSCT) is not able to improve survival of these patients. A similar observation has been made in patients with MDS or s-AML harboring more than two unfavorable molecular genetic aberrations (Yoshizato et al. 2017).

AML following previous chemotherapy or radiation treatment (t-AML) can be regularly observed in patients with history of lymphoma (e.g., Hodgkin’s disease) or breast cancer (Bertoli et al. 2016; Linassier et al. 2000). Cytogenetic analyses of t-AML reveal aberrations–e.g., t(8;21) or inv(16)—that are associated with a good prognosis in de novo AML according to the ELN classification (Gustafson et al. 2009; Döhner et al. 2017). Importantly, such cytogenetic findings cannot be attributed to an improved outcome in t-AML patients (Gustafson et al. 2009; Schoch et al. 2004). Furthermore, NPM1 mutations and FLT3 internal tandem duplication (FLT3-ITD) are less frequently detected in patients with t-AML while regularly identified TP53 mutations are associated with a lower response rate to conventional chemotherapy (Kayser et al. 2011). Surprisingly, progenitor cells harboring age-related TP53 mutations seem to be enriched during chemotherapy leading to the worse prognosis of TP53 mutated t-AML patients (Wong et al. 2015).

Induction chemotherapy of elderly AML patients is regularly associated with a lower response rate due to a higher proportion of patients with high-risk characteristics (e.g., antecedent MDS, cytogenetic risk factors) (Bello et al. 2011). A higher rate of early death following induction chemotherapy because of treatment-related complications (e.g., severe infections) has been observed (Krug et al. 2010). Improvement of induction chemotherapy approaches to achieve a better response rate without increased toxicity is an unmet medical need in elderly high-risk AML patients. Intensification of chemotherapy (e.g., induction treatment according to the FLAG protocol) has been demonstrated to improve complete remission rate without increasing mortality in patients with secondary AML (Vulaj et al. 2018). In contrast, the addition of the CD33-directed monoclonal antibody–drug conjugate gemtuzumab-ozogamicin had no clinically relevant impact on the outcome of AML patients with high-risk cytogenetics (Castaigne et al. 2012).

Several clinical trials addressed the question to improve chemotherapy regimens for AML patients. The OSHO study group (East German Study Group of Hematology and Oncology) has implemented intermediate-dosed cytarabine in different age-dependent induction protocols. For younger patients (up to the age of 60 years), response rate and survival of AML patients has been demonstrated to be similar compared to conventional induction regimens (e.g., 7 + 3). Furthermore, there is a substantial benefit in elderly AML patients (60–75 years) after OSHO induction therapy followed by allo-HSCT (Büchner et al. 2012; Niederwieser et al. 2014).

Long-term outcome of high-risk AML is highly dependent on the eligibility to allo-HSCT while the introduction of reduced-intensity conditioning (RIC) regimens allows allo-HSCT in patients up to the age of about 75 years. Large registry analyses could not demonstrate any difference in overall survival of AML and MDS patients comparing myeloablative conditioning (MAC) and RIC regimens (Luger et al. 2012). In the context of the increasing risk of treatment-related mortality (TRM) in patients between the age of 60 and 75 years, the role of RIC regimens has been established and can reduce TRM in elderly AML patients (Ustun et al. 2019; Tauro et al. 2005).

The liposomal formulation of cytarabine and daunorubicin (CPX-351, Vyxeos^®^) has been approved for induction and consolidation chemotherapy for patients with AML-MRC or t-AML by the US Food and Drug Administration (FDA) and the European Medicine Agency (EMA). CPX-351 has been evaluated in a phase 3 clinical trial in patients with AML-MRC or t-AML undergoing induction chemotherapy with either 7 + 3 regimen (cytarabine plus daunorubicin) or CPX-351 (Lancet et al. 2018).

There was a higher response rate in the CPX-351 cohort as compared to standard 7 + 3 induction chemotherapy resulting in a significant longer overall survival (9.6 vs 6.0 months). Patients subsequently undergoing allo-HSCT demonstrated the clinically most relevant benefit with a survival rate of about 60% two years after allo-HSCT. Recently, these data have been confirmed in a large cohort of 109 high-risk AML patients in France (Chiche et al. 2021).

Here, we report on a retrospective analysis of 110 consecutively treated AML patients with s-AML or t-AML. All these patients were treated with induction chemotherapy containing intermediate-dosed cytarabine (ID-AraC) in combination with either idarubicin or mitoxantrone while most patients underwent subsequent allo-HSCT in first complete remission.

## Patients and methods

### Patient cohort

**Fig. 1:**
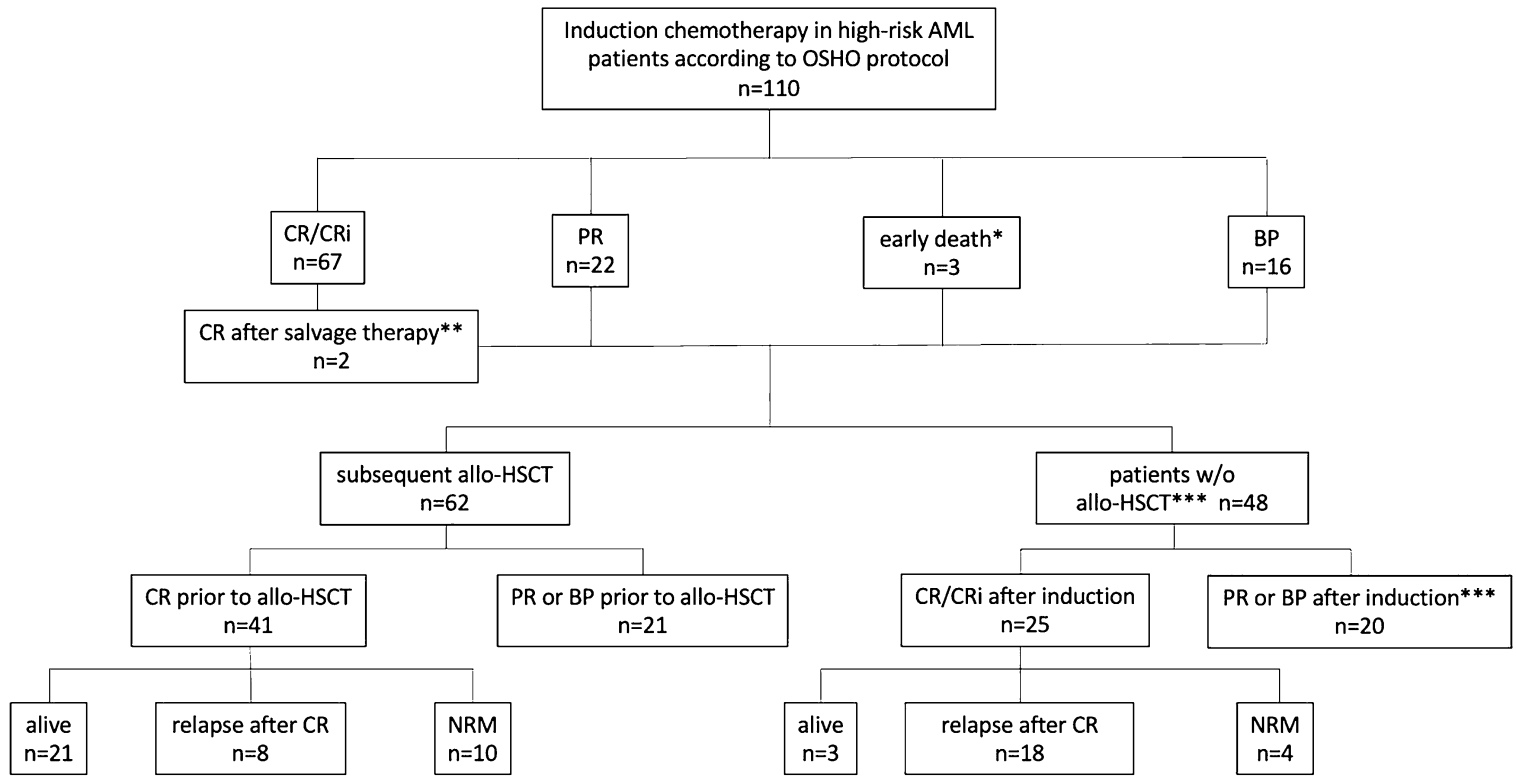
CONSORT diagram response to induction therapy and follow- up after CR. *Including two pts who died until day 30 and one patient who died on day 44 after start of induction chemotherapy. **Both patients received salvage chemotherapy according to Mito-FLAG protocol, one of these pts subsequently underwent allo-HSCT. *** 1 pt died during consolidation after CR; 4 pts died during consolidation after PR/BP

A total of 110 consecutively treated patients with either s-AML or t-AML were identified. All these AML patients underwent induction chemotherapy at the Department of Hematology and Oncology, University Hospital Jena, Germany. Diagnosis and start of induction chemotherapy were between 2001 and 2020. The cohort consisted of 23 patients (20.9%) with t-AML including 8 patients with a history of lymphoma treatment and 11 women who developed t-AML following chemotherapy due to breast cancer. Patients´ characteristics are indicated in Table 1. Figure 1S (Supplement) provides clinical details and time course of individual history prior to diagnosis of t-AML.

**Table 1:** Patient’s characteristics

Patients ‘characteristics, *n* = 110	*n* (%)
Sex female	58 (53)
Median age, years [range]	62 [24–77]
No. with WBC at diagnosis, > 20 × 10^9^/l ≤ 20 × 10^9^/l	36 (32.7) 74 (67.2)
Platelet count at diagnosis, median [range], × 10^9^/l	56 [4–738]
Hemoglobin level at diagnosis, median [range], mmol/l	5.6 [3.7–9.3]
Peripheral blast count at diagnosis, median [range]	12 [0–97]
Bone marrow blasts at diagnosis, median [range]	40 [5–98]
AML subtype	
t-AML	23 (20.9)
s-AML after MDS	65 (59)
s-AML after CMML	15 (13.6)
s-AML after MPN	7 (6.3)
HMA prior to induction	28 (25.4)
FLT3 and NPM1 mutation status	
FLT3-ITD mutation	10 (14.9)
FLT3 wild-type	57 (85.1)
* Missing*	43 (39)
NPM1 mutation	4 (8.8)
NPM1 negative	41 (91.2)
* Missing*	65 (59)
FAB subtype	
M0	2 (1.8)
M1/M2	65 (59)
M4/M5	35 (31.8)
M6	8 (7.2)
Cytogenetic abnormalities	
Normal karyotype	46 (41.8)
Complex aberrant	22 (20)
t(8;21) and inversion 16	4 (3.6)
Trisomy 8	8 (7.2)
Monosomal karyotype	9 (8.1)
MLL-rearrangement	2 (1.8)
Other	14 (12.7)
n.d	5 (4.5)
Cytogenetic risk	
Favorable	3 (2.7)
Intermediate	69 (62.7)
Adverse	33 (30)
n.d	5 (4.5)
ELN risk 2010 (in 42/110 with both available NPM1 and FLT3 mutational status)	
Favorable	4 (9.5)
Intermediate (I + II)	28 (66.6)
Adverse	10 (23.8)
n.d	68 (61.8)

*t-AML* therapy associated acute myeloid leukemia, *s-AML* secondary AML, *MDS* myelodysplastic syndrome, *CMML* chronic myelomonocytic leukemia, *MPN* myeloproliferative syndrome, *n.d.* not determined, *ELN* European leukemia Net, *WBC* white blood count, *HMA* hypomethylating agent, *GPT/l* gigaparticle per liter

### Informed consent

All patients were included in one of the following AML registries: AML registry of the OSHO study group (East German Study Group of Hematology and Oncology) or in the SAL registry (Study Alliance Leukemia). Patients gave their written informed consent for data acquisition and analysis after pseudonymization in one of the AML registries. The participation in both AML registries has been approved by the Ethical review committee of the University Hospital Jena (Ethical number 4871–07/16 for “retrospective evaluation of therapy response and survival in patients with AML” and 3967–12/13 for SAL registry).

### Patient treatment

Induction and consolidation chemotherapy was applied according to the OSHO 2002 (NCT01414231) or the OSHO 2004 (NCT01497002) protocol. In detail, patients up to the age of 60 years were treated with idarubicin (12 mg/m^2^, day 1–3) and intermediate-dosed cytarabine (1 g/m^2^ bid, day 1, 3, 5 and 7) as induction chemotherapy while patients over 60 years received mitoxantrone (10 mg/m^2^, day 1–3) and intermediate-dosed cytarabine (1 g/m^2^ bid, day 1, 3, 5 and 7) as induction treatment. First consolidation chemotherapy in younger AML patients was identical with induction chemotherapy. Elderly AML patients underwent consolidation treatment with a dose reduction of mitoxantrone (10 mg/m^2^, day 1 and 2) and intermediate-dosed cytarabine (0.5 g/m^2^ bid, day 1, 3 and 5) (Büchner et al. 2012; Kahl et al. 2016).

The majority of patients (47/62, 75.8%) subsequently undergoing allo-HSCT received a reduced-intensity conditioning (RIC) based on treosulfan (36/47, 76.6%) or busulfan (8/47, 17.0%) in combination with fludarabine prior to allo-HSCT (Casper et al. 2012; Kröger et al. 2003). In addition, three patients (6.4%) of the RIC subgroup received the FLAMSA protocol before allo-HSCT (Schmid et al. 2008). The remaining patients underwent either myeloablative conditioning (MAC; 10/62, 16.1%) with 12 Gy total body irradiation (TBI) in combination with cyclophosphamide or a non-myeloablative conditioning (NMAC; 5/62, 8.1%) with 2 Gy TBI and fludarabine (Jethava et al. 2017; Niederwieser et al. 2003).

Graft-versus-host-disease (GvHD) prophylaxis consisted of cyclosporine A (CSA) in combination with short-term methotrexate (MTX) or mycophenolate mofetil (MMF), and anti-thymocyte globulin (ATG). Patient characteristics of those patients who subsequently underwent allo-HSCT are summarized in Table 4. Calculation of comorbidity score prior to allo-HSCT was performed according to Sorror (Sorror et al. 2005).

### Analysis of toxicity and classification of fungal infections

Toxicity during and after induction chemotherapy was evaluated according to the Common Terminology Criteria and Adverse Events classification (CTCAE v4.0). Fungal infections have been defined according to the EORTC/MSG nomenclature classifying possible, probable and proven invasive fungal disease (IFD) (Pauw et al. 2008).

### Cytogenetic and molecular genetic analyses

Karyotype analyses by means of chromosome banding were performed with standard techniques, and karyotypes were described according to the International System for Human Cytogenetic Nomenclature (Brothman et al. 2005). Cytogenetic categorization into favorable, intermediate or adverse risk was performed on the basis of recommended criteria (Döhner et al. 2017).

In all patients with chromosomal aberrations, detection of residual AML cells was established using fluorescence in situ hybridization (FISH) and applied after induction chemotherapy and prior to allo-HSCT. A summary of cytogenetic remission status at particular time points is shown in the supplement (Table S2).

The presence of FLT3-ITD and NPM1 mutations was detected by PCR amplification of the corresponding region using genomic DNA followed by fragment analysis (Scholl et al. 2008,2005).

### Statistics

Determination of complete remission (CR) without or with incomplete recovery (CRi), partial remission (PR) and refractory disease (persistence of blasts) was performed according to the current guidelines of the European Leukemia Network (ELN 2017). Overall survival (OS), Event-free survival (EFS) and relapse-free survival (RFS) were also defined using the ELN 2017 recommendations (Döhner et al. 2017). For patients undergoing allo-HSCT, OS and RFS were additionally calculated from the date of allo-HSCT to the date of death.

Statistical analyses were performed using GraphPad Prism 8.0.2 (GraphPad Inc.). Differences between the Kaplan–Meier survival curves were evaluated by Log-rank (Mantel-Cox) test. *p* values of < 0.05 were considered as statistically significant.

## Results

### Patient’s characteristics

This analysis comprises 110 patients with a median age of 62 years (range 24–77) who received intensive AML-treatment. The majority of patients (*n* = 65, 59%) developed AML following antecedent MDS, 15 patients (13.6%) after chronic myelomonocytic leukemia (CMML) and 7 patients (6.3%) following myeloproliferative syndrome (MPN). Detailed clinical characteristics and time course of 23 patients (20.9%) with t-AML are illustrated in Table S1 and Figure S1, respectively. 28 patients (25.4%) received treatment with hypomethylating agents prior to induction chemotherapy.

In 67 (43%) and 45 cases (40.9%) with available data, FLT3-ITD or NPM1 mutations could be detected in 10 (14.9%) and 4 patients (8.8%), respectively. According to ELN risk classification 2010, risk score was favorable, intermediate (I or II) and adverse in 4 patients (9.5%), 28 patients (66.6%) and 10 patients (23.8%), respectively. In 68 cases (61.8%) no stratification was possible due to lack of mutational testing. Therefore, additional risk stratification based on cytogenetic data only is demonstrated in Table 1. An overview of our study population including subsequent allo-HSCT in the majority of patients is given in the CONSORT diagram (Fig. 1).

### Response to induction chemotherapy

**Fig. 2:**
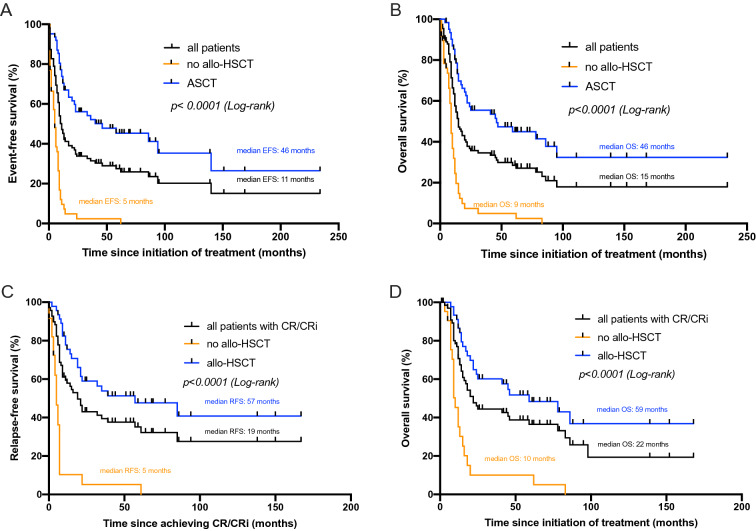
Kaplan–Meier estimates for EFS (**A**), OS (**B**, **D**) and RFS (**C**). **A** comparison between EFS of whole cohort vs. allo-HSCT vs. no allo-HSCT. **B** comparison of OS of whole cohort vs. allo-HSCT vs. no allo-HSCT. **C** RFS for patients achieving CR or CRi is shown for all patients vs. allo-HSCT vs. no allo-HSCT. **D** OS for patients achieving CR or CRi is demonstrated for all patients vs. allo-HSCT vs. no allo-HSCT

The analysis of overall remission after induction chemotherapy revealed a CR/CRi rate of 62.7% (69 of 110 patients). In 21% (23/110) a PR was observed, 11.8% (13/110) were refractory to induction therapy. In two of these refractory patients, CR could be achieved after salvage therapy by Mito-FLAG protocol. One of both patients further proceeded to allo-HSCT (Fig. 1). The median OS of the whole cohort was 15 months (0–234) and the median EFS was 11 months (0–234) while the median RFS for patients who achieved CR/CRi was 19 months (0–167; all *p* < 0.0001, Fig. 2). The median follow-up time of all 110 patients was 13 months (1–234). Additionally, cytogenetic remission (FISH) following induction treatment as well as prior to allo-HSCT is shown in Table S2 in the supplement.

### Safety and hematological recovery

During induction, early deaths occurred in 3 (2.7%) patients including 2 patients dying within the first 30 days after start of induction chemotherapy and one patient who died on day 44 in aplasia during the course of induction (Table 2). The 90-day mortality after induction chemotherapy was 7.2% (8/110).

**Table 2:** Outcome after induction chemotherapy

Outcome after induction chemotherapy *n* = 110	*n* (%)
OSHO < 60 years	36 (32.7)
OSHO > 60 years	74 (67.2)
Response rate after induction therapy	
CR/CRi	69 (62.7)
PR	23 (20.9)
Refractory AML	13 (11.8)
CR after salvage therapy	2 (1.8)
Not performed	3 (2.7)
Hematological reconstitution	
G-CSF support	23 (20.9)
Platelets > 50 × 10^9^/l, median days [range]	29 [17–122]
Platelets > 50 × 10^9^/l, only CR, median days [range]	30 [17–90]
Not achieved	13 (11.8)
WBC > 2.0Gpt/l or neutrophils > 0,5 × 10^9^/l, median days [range]	30 [21–141]
WBC > 2.0Gpt/l or neutrophils > 0,5 × 10^9^/l, only CR, median days [range]	30 [21–141]
Not achieved	11 (10)
Median follow-up, months (range)	13 (1–234)
30-day mortality	2 (1.8)
90-day mortality	8 (7.2)

*OSHO* Ostdeutsche Studiengruppe für Hämatologie/Onkologie, *CR* complete remission, *CRi* complete remission with incomplete hematological recovery, *PR* partial remission, *G-CSF* granulocyte colony-stimulating factor, *Gpt/l* gigaparticle per liter, *OS* overall survival

In general, analysis of non-hematologic toxicity showed moderate side effects during induction chemotherapy. In Table 3, non-hematologic toxicity according to CTC classification is listed. Among grade III and IV toxicities, five patients (4.5%) occurred with severe pneumogenic sepsis with need of intensive care treatment. One patient presented with seizures, another patient suffered from heart attack. Classification of invasive fungal disease (IFD) indicated invasive fungal disease in 38 patients (35%). Probable or proven IFD could be documented in only 4.5 and 3%, respectively.

**Table 3:** Non-hematologic toxicity and IFD during induction chemotherapy

Non-hematologic toxicity, *n* = 110	I–II	III–IV
Diarrhea	5 (4.5)	0
Nausea/vomiting	3 (2.7)	4 (3.6)
Impaired heart function	3 (2.7)	3 (2.7)
Mucositis	10 (9.0)	3 (2.7)
Bilirubin elevation	26 (23.6)	1 (0.9)
ASAT/ALAT elevation	34 (30.9)	10 (0.9)
Skin reaction	11 (10.0)	0
Creatinine increase	13 (11.8)	1 (0.9)
Neurological events	0	1 (0.9)
Fever in neutropenia	0	99 (90)
Pneumonia	0	59 (53)
Sepsis with need of ICU	0	5 (4.5)
Invasive fungal disease (%)	38 (35.0)	
Possible	30 (27.2)	
Probable	5 (4.5)	
Proven	3 (2.7)	
No IFD	72 (65.0)	

*IFD* invasive fungal disease, *ICU intensive care unit*

The assessment of hematological reconstitution after induction chemotherapy showed a median time of platelet recovery of at least 50 × 10^9^/l after 29 days (17–122 days) and a median time of white blood count (WBC) recovery > 2.0 × 10^9^/l of 30 days or absolute neutrophil counts > 0.5 × 10^9^/l (21–141 days). 11.9% (13/110) and 10% (11/110) did neither achieve stable reconstitution of platelets or neutrophils (Table 2). Notably, 23 patients (20.9%) received granulocyte colony-stimulating factor (G-CSF) support to accelerate neutrophil recovery in cases of delayed hematologic reconstitution.

### Outcome and safety after allogeneic SCT

In total, 62 patients (56.3%) proceeded to allo-HSCT including 66.1% (41/62) in first CR. 15 (24.1%) patients fulfilled criteria for PR and 6 (9.6%) patients had a relapsed or refractory AML at the time of transplantation. The median age at transplantation was 58 years (24–73 years).

Kaplan–Meier analysis showed a median OS of patients who received allo-HSCT of 46 months (0–234 months) vs. 9 months (0–98 months) without allo-HSCT, *p* < 0.0001. After a median follow-up time of 25.5 months (4–234 months) since diagnosis of AML and 22 months (3–230 months) since date of transplantation, RFS could demonstrate a statistically significant benefit of 57 months (1–167 months) for patients with allo-HSCT compared to 5 months (0–61 months) for those patients who did not underwent allo-HSCT, *p* < 0.0001 (Table 4). Median EFS was 46 vs. 5 months for patients with or without allo-HSCT, respectively.

**Table 4:** Clinical characteristics and outcome after allo-HSCT

Clinical characteristics and outcome after allo-HSCT, *n* = 62	*n* (%)		
Allo-HSCT	62/110 (56.3)		
Age at transplantation [range]	58 [24–73]		
Conditioning regime			
RIC	47 (75.8)		
* Treosulfan/Fludarabin/ATG*	36 (76.6)		
* Treosulfan/Busulfan/ATG*	8 (17)		
* FLAMSA*	3 (6.4)		
MAC	10 (16.1)		
NMAC	5 (8)		
Remission prior to allo-HSCT			
CR1	41 (66.1)		
PR	15 (24.1)		
Relapsed/refractory	6 (9.6)		
Donor compatibility			
mRD	12 (19.3)		
mUD	36 (58)		
mmUD	14 (22.5)		
Sorror Score, points (%)	**0**	**1/2**	** ≥ 3**
RIC	14 (29.7)	11 (23.4)	22 (46.8)
MAC	7 (70)	2 (20)	1 (10)
NMAC	0	2 (50)	2 (50)
Median follow-up since allo-HSCT [range]	22 [2–230]		
30-day mortality	1 (1.6)		
90-day mortality	2 (3.2)		
NRM after allo-HSCT Median days [range]	18 (29) 234.5 [22–2766]		
Sepsis	14/18 (77.7)		
Other	4/18 (22.2)		

*allo-HSCT *allogeneic stem cell transplantation, *RIC *reduced-intensity conditioning,* ATG *anti-thymocytic globulin; *MAC *myeloablative conditioning, *NMAC *non-myeloablative conditioning, *mRD *matched related donor, *mUD *matched unrelated donor, *mmUD *mismatched unrelated donor, *NRM *non-relapse mortality

The majority of 58% (36/62) received a matched, unrelated graft (MUD). Compared with the group of patients who obtained matched related or mismatched unrelated grafts there was a trend towards a higher RFS for patients receiving a MUD graft (data not shown, *p* = 0.1209).

Two patients (3.2%) died within the first 90 days following allo-HSCT, both on septic complications. Assessment of non-relapse mortality after allo-HSCT identified 18 patients. These died after a median of 235 days (22–2766 days) post allo-HSCT with major cause of sepsis and severe infections.

### Impact of conditioning regimen on survival after allo-HSCT

**Fig. 3:**
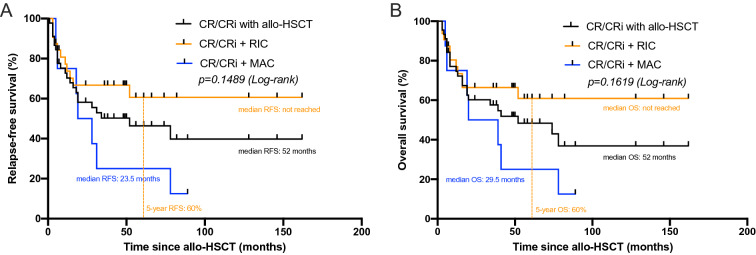
Kaplan–Meier estimates for RFS (**A**) and OS (**B**) demonstrating the impact of conditioning regimen prior to allo-HSCT on survival. Both curves are calculated since date of allo-HSCT for patients who achieved CR/CRi prior to allo-HSCT

The vast majority of patients with allo-HSCT (75.8%, 47/62) received RIC protocols prior to allo-HSCT. The comparison of OS between patients who received either RIC or MAC prior to allo-HSCT demonstrated a statistically non-significant advantage in survival for patients following RIC conditioning. The median OS for patients in CR/CRi who received MAC conditioning was 29.5 months, for RIC group OS was about 60% after 60 months post allo-HSCT (median OS not reached, Fig. 3B). In accordance with that, RFS for all patients with CR/CRi prior to transplantation was 52 months, for the MAC conditioned patients 23.5 months while median RFS for the RIC group has not been reached (*p* = 0.1489, Fig. 3A). comparison of 5-year OS of patients who did not undergo allo-HSCT following CR/CRi after induction chemotherapy with those receiving allo-HSCT with RIC elucidated a clinically relevant difference of 9.5 vs. 60%, respectively.

Risk assessment prior to allo-HSCT according to “Sorror” comorbidity score displayed an extend of comorbidities in the RIC conditioned cohort, while MAC conditioned patients showed much lower comorbidity levels (Table 4).

## Discussion

Treatment of AML remains a challenge in both younger and elderly AML patients and there is an increased risk of negative prognostic factors correlating with patients´ age at diagnosis. Antecedent myelodysplastic syndrome (MDS) or de novo AML with myeloid-related changes (AML-MRC) is especially seen in elderly patients which makes an evaluation of the eligibility of allogeneic stem cell transplantation (allo-HSCT) essential.

Recently, clinically relevant improvement of AML therapy could only be achieved either for distinct molecular or cytogenetic subgroups (e.g., midostaurin in FLT3 mutated AML or gemtuzumab ozogamicin for AML with favorable prognosis) or for patients with relapsed/ refractory AML harboring activating FLT3 mutations (e.g., gilteritinib) to replace intensive salvage chemotherapy (Castaigne et al. 2012; Stone et al. 2017; Perl et al. 2019).

Induction chemotherapy of high-risk AML patients with a conventional 7 + 3 regimen has been the standard of care in many AML study groups. The approval of liposomal chemotherapy combining cytarabine and daunorubicin (CPX-351) is currently changing this standard induction chemotherapy for a wide range of high-risk AML patients. Here, a comprehensive monocentric analysis of induction chemotherapy with intermediate-dosed cytarabine according to two well-established and age-adjusted protocols that have been intensively evaluated within the OSHO group is presented.

The distribution of distinct prognostic subgroups according to cytogenetic findings reveals an expected percentage of patients with unfavorable cytogenetics while there are only few patients that can be attributed to the favorable risk subgroup. This observation is in line with several other reports investigating patients with s-AML or t-AML and does also include a relatively low percentage of NPM1 or FLT3 mutations as opposed to younger AML patients with de novo AML. A relevant limitation of our retrospective study is the incomplete data set for molecular genetics (e.g., ASXL1, RUNX1 or TP53) to apply ELN 2017 classification. Thus, we could only stratify our cohort of AML patients according to cytogenetic risk groups with a potential higher percentage of patients attributed to the intermediate risk group. Nevertheless, the whole patient cohort analyzed here comprises high-risk AML patients with either s-AML or t-AML.

Following induction therapy with intermediate-dosed cytarabin an excellent rate of complete remission is demonstrated in this cohort of high-risk AML patients indicating a potential benefit for intermediate-dosed cytarabine compared to its application by continuous infusion. In consideration of previously published clinicals trials evaluating high-dosed cytarabine for induction chemotherapy, we hypothesize that the distinct pharmacokinetic of intermediate-dosed cytarabine as compared to the 7 + 3 regimen might be more effective to overcome primary resistance (Reese and Schiller 2013).

Furthermore, intermediate-dosed cytarabin chemotherapy is associated with a low rate of early death. In detail, only five patients developed sepsis with the need of intensive care treatment and the rather low rate of proven or probable invasive fungal infections might be a result of effective antifungal prophylaxis with posaconazole in the majority of patients.

In this cohort of high-risk AML patients with comparable characteristics concerning the percentage of secondary AML and t-AML patients’ reconstitution of neutrophils and platelets following both OSHO regimens applying intermediate-dosed cytarabine was in time. Importantly, 23 (20.9%) patient received granulocyte colony-stimulating factor (G-CSF) to accelerate neutrophil recovery.

Hematologic reconstitution after induction chemotherapy with CPX-351 is significantly delayed compared with the conventional 7 + 3 induction regimen. The differences in time to recovery of neutrophils and platelets are explained by distinct pharmacokinetic properties of CPX-351 with prolonged exposure to co-encapsulated cytarabine and daunorubicin in this liposomal formulation (Lancet et al. 2018).

The median age of patients in our cohort of high-risk AML patients was about five years below the median age of patients treated with CPX-351 published within the phase 3 trial or in the French AML survey (Lancet et al. 2018; Chiche et al. 2021). This might be one explanation for the lower rate of consolidation treatment with allo-HSCT in both studies (29 vs 35%, respectively). In our study a rather high rate of patients underwent allo-HSCT as recommended for this subset of AML patients being characterized by a high risk of AML relapse especially without allo-HSCT consolidation.

The present data demonstrate the impact of allo-HSCT on survival of high-risk AML patients in first complete remission. This reflects the high relevance of such immunotherapeutic approaches as allo-HSCT for patients with s-AML or t-AML. In general, we could show a low rate of early mortality after allo-HSCT in our patient cohort. While only two patients died of transplantation-related complications until day 90 after allo-HSCT, TRM had to be documented in another 16 patients within a long time period following allo-HSCT. This observation reflects the generally high vulnerability of immunocompromised patients in the context of allo-HSCT that can be associated with TRM even several years after allo-HSCT (Shimoni et al. 2016).

In consideration of age and comorbidities, there was a high rate of patients who received a reduced-intensity conditioning (RIC) regimen prior to allo-HSCT. The development of RIC protocols has substantially improved the eligibility of elderly AML patients for allo-HSCT as a basis to increase the survival of high-risk AML patients. Despite a comparable survival rate for patients undergoing MAC or RIC protocols about two years after allo-HSCT, there is a clinically relevant higher and stable long-term overall survival in the subgroup of patients who underwent RIC prior to allo-HSCT. This emphasizes the clinical benefit of RIC regimens for high-risk AML patients with the potential to cure AML (Casper et al. 2012).

Despite a higher percentage of patients with secondary AML or t-AML can be attributed to the ELN subgroup with an adverse prognosis, outcome of these patients after allo-HSCT is comparable with de novo AML patients of this ELN subgroup (Jentzsch et al. 2020). Furthermore, RIC regimens prior to allo-HSCT can significantly reduce TRM improving the outcome of patients with high-risk AML (Tauro et al. 2005). For patients with normal karyotype t-AML, Samra and colleagues could demonstrate a significantly higher rate of death in remission but no higher relapse rate following induction chemotherapy and allo-HSCT (Samra et al. 2020). This supports our hypothesis that RIC prior to allo-HSCT has a high impact on long-term survival in patients with secondary AML or t-AML.

TP53 mutations are considered to play a key role in mediating resistance to conventional chemotherapy. Furthermore, Walter et al. could show that further mutations (e.g., PTPN11) might contribute to primary resistance of secondary AML. Previously published real-life experience of CPX-351 in a cohort of 103 French high-risk AML patients could confirm the inferior outcome of those patients harboring TP53 or PTPN11 mutations (Chiche et al. 2021; Walter et al. 2012).

Molecular characterization of high-risk AML helps the clinicians to identify patients with potentially refractory AML following conventional induction chemotherapy. This is not only clinically relevant in patients with a reduced performance status resulting in a higher risk of severe morbidity or even early death after induction chemotherapy. Recently established improvement of epigenetic therapy by combination with the BCL-2 inhibitor venetoclax or targeted therapy in IDH1 or IDH2 mutated AML offers reasonable therapeutic option with a significantly lower risk of severe complications as compared to intensive chemotherapy regimens (DiNardo et al. 2020; Roboz et al. 2020; Pollyea et al. 2019).

Induction therapy with intermediated-dose of cytarabine results in comparable rate of complete remission rates with CPX-351. A major finding of this study was the demonstration of a significantly longer overall survival of patients undergoing allo-HSCT as consolidation treatment. Despite the latter aspect is independent of the induction regimen, our data on overall survival following OSHO induction chemotherapy and allo-HSCT reflects a promising approach for this challenging cohort of high-risk AML patients.

Taken together, induction chemotherapy with intermediate-dosed cytarabine according to the OSHO protocols followed by allo-HSCT provides a reasonable strategy for treatment of high-risk AML patients.

## Supplementary Information

Below is the link to the electronic supplementary material.

Supplementary file1 Fig. S1 Clinical details and time course of patient’s history prior to diagnosis of t-AML (PDF 298 KB)

Supplementary file2 (DOCX 16 KB)
