# Supplementary figures and images for: Impact of induction chemotherapy with intermediate-dosed cytarabine and subsequent allogeneic stem cell transplantation on the outcome of high-risk acute myeloid leukemia
Source: J Cancer Res Clin Oncol. 2021 Jul 23;148(6):1481–92. doi: 10.1007/s00432-021-03733-0 (PMC9114033; doi:10.1007/s00432-021-03733-0)

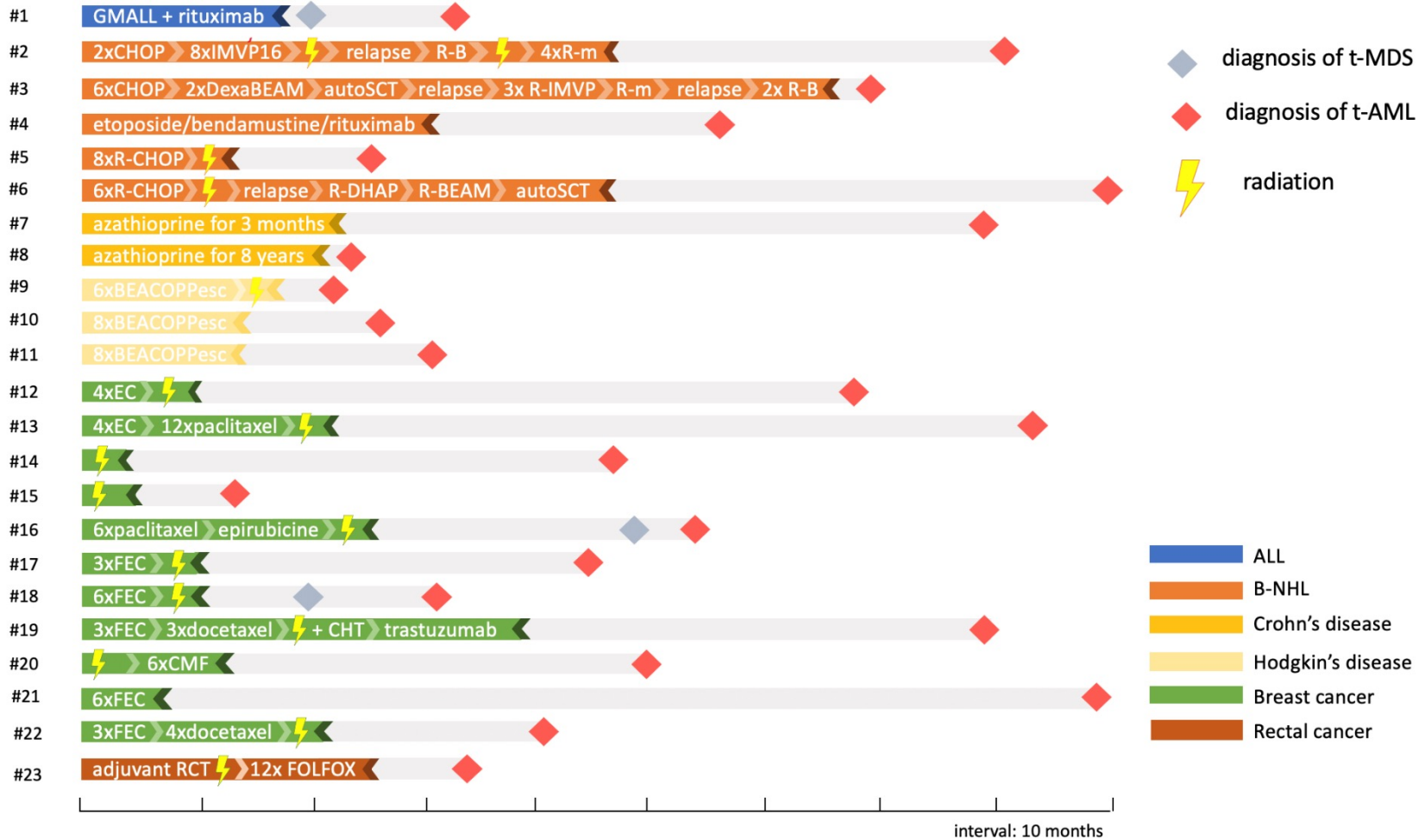

Supplement: Supplementary file 1 — Supplementary file1 Fig. S1 Clinical details and time course of patient’s history prior to diagnosis of t-AML (PDF 298 KB) [file 432_2021_3733_MOESM1_ESM.pdf]
