# Supplementary material for: Impact of induction chemotherapy with intermediate-dosed cytarabine and subsequent allogeneic stem cell transplantation on the outcome of high-risk acute myeloid leukemia
Source: J Cancer Res Clin Oncol. 2021 Jul 23;148(6):1481–92. doi: 10.1007/s00432-021-03733-0 (PMC9114033; doi:10.1007/s00432-021-03733-0)
Supplement: Supplementary file 2 — Supplementary file2 (DOCX 16 KB) [file 432_2021_3733_MOESM2_ESM.docx]

| Subgroup of t-AML, n=23 | n (%) |
| --- | --- |
| **Primary disease** |  |
| Non-hodgkin lymphoma | 5 (21.7) |
| Hodgkin’s disease | 3 (13) |
| Breast cancer | 11 (47.8) |
| Acute lymphatic leukemia | 1 (4.3) |
| Crohn's disease | 2 (8.6) |
| Rectal cancer | 1 (4.3) |
| Median time from end of primary treatment to AML onset, month [range] | 30 [0-100] |

**Table S1**: Overview about antecedent primary diseases before AML onset.

| Cytogenetic remission of pts with CR/CRi following to induction chemotherapy, n=68 | n (%) |
| --- | --- |
| FISH applicable | 37 (54.4) |
| Results available | 25/37 (67.5) |
| CR | 22/25 (88) |
| PR | 3/25 (12) |
| **Cytogenetic remission of pts with CR/CRi prior to allo-HSCT** | 41/62 (66.1) |
| FISH applicable | 23/41 (56) |
| Results available | 18/23 (78.2) |
| CR | 14/18 (77) |
| PR | 4/18 (22) |

**Table S2:** Assessment of cytogenetic remission status after induction chemotherapy and prior to allo-HSCT in patients who achieved CR/CRi (when exhibiting usable cytogenetic markers).
